# First Report of Known Rare Rhnull Phenotype Individuals in Iran

**Published:** 2018-07-01

**Authors:** Ehsan Shahverdi, Mostafa Moghaddam, Hassan Abolghasemi

**Affiliations:** 1 Blood Transfusion Research Center, High Institute for Research and Education in Transfusion Medicine, Tehran, Iran; 2 Pediatric Congenital Hematologic Disorders Research Center, Shahid Beheshti University of Medical Sciences, Tehran, Iran

**Keywords:** Rhnull, Blood group, Hemolytic anemia, Antibody screening, Iran

## Abstract

Rhnull phenotype is a rare blood group with a frequency of approximately 1 in 6 million individuals, transmitted via an autosomal recessive mode. It is characterized by the weak (Rhmod) or lack (Rhnull) of expression of all Rh antigens on the red cells. The clinical significance of its assessment is that such patients with Rhnull syndrome are associated with chronic hemolytic anemia of varying degrees. Another clinical importance is that such subjects readily form alloantibodies when exposed to Rh antigens.We report herein a rare Rhnull phenotype in a sibling, which was detected as a part of the difficult sample work-up for red cell antibody screening and identification.

## Introduction

 The Rh-deficiency syndrome is a rare genetic disorder of red blood cells (RBCs) with a reported frequency of approximately 1 in 6 million individuals, transmitted via an autosomal recessive mode generally via consanguineous genealogy ^1^^,^^2^ . The characteristic hallmark of Rhnull phenotype is the lack of all Rh antigens on the RBCs. The Rhnull patients manifest a mild to moderate hemolytic anemia, and their RBCs show changes in morphology (stomatocytosis) and abnormalities in plasma membranes ^3^^-^^6^ . It results in the same clinical syndrome characterized by chronic hemolysis of varying severities, with stomatocytosis, spherocytosis, increased osmotic fragility, altered phospholipids asymmetry, altered cell volume, defective cation fluxes, and elevation in Na+/K+ ATPase activity^   2 ^. 

To the best of our knowledge, this is the first report about individuals with identified Rhnull syndrome across Iran.

## Case presentation

A-43-year old female with severe anemia following splenectomy previously typed as blood group A, Rh (D) negative, was referred to Immunohematology Reference Laboratory (IRL) of the Iranian Blood Transfusion Organization (IBTO)*,* Tehran, Iran for ABO / Rh(D) typing and antibody screening test with a request for two units of RBC for transfusion in December 2013. Patient’s medical history revealed recurrent abortion and miscarriage with no history of blood transfusion. Her family history revealed that her parents had a consanguineous marriage and she had four siblings, all were alive without any suspected blood disorder, except one of the brothers that underwent splenectomy because of hereditary spherocytosis anemia. Her only previous child was a healthy fifteen-year-old boy, who was typed and was not confirmed as Rhnull phenotype. Coagulation and hematology parameters were in the normal range, except for very low hemoglobin of 3.2 g/dL. 

It was observed that the patient’s serum plasma reacted strongly in antibody panel cells, giving 4 + macroscopic in 37℃ phase and in the anti-human globulin phase. Auto control test result was negative. Direct Anti-globulin Test (DAT) was positive (1^+^) with differential anti- IgG negative and anti-C_3_d positive (1^+^). 

These results suggested the presence of clinically significant alloantibodies against multiple negative antigens or a high-prevalence antigen. An antibody screening test result was negative for the patient’s brother. A home-made available three-cell antigen panel (IBTO mini-panel) was used for the antibody screening procedure in which the patient's plasma was added to RBCs without papain enzyme using the Low Ionic Strength Saline (LISS). IBTO mini-3cell panel and antibody identification 11cell kit and also selected cells were validated within the two-year period using commercial CE marked Diamed kits. The antibody screening test was performed twice in parallel using IBTO produced kits and Diamed kits. The results were compared and in case of positive results, the 11cells antibody ID panel from Diamed Company was used simultaneously with IBTO 11cells antibody ID panel. IBTO homemade antibody ID panel and selected cells were used to exclude and include alloantibodies.

Column agglutination method with antiglobulin gel card (INVITROGEL AHG coombs-Germany) was used for the antibody screening test. The gel cards were incubated at 37 °C for 15 minutes, and then centrifuged for 10 minutes. Standard tube methods (Bio-Rad AHG- Germany) were used for antibody identification and selected cell tests. 

Clinically significant alloantibodies were defined as those antibodies that potentially could cause RBC destruction based on the reactivity at 37 °C and/or anti-human globulin (AHG) phase. 

The patient and her brother extended phenotyping showed that they were negative for D, C, E, c, e RBC antigens, indicating they were strongly suspicious of being the rare Rhnull phenotype.

Adsorption and elution studies in Anti-human globulin (AHG, CE- Immunodiagnostika, Am Seerain 13 Germany, Eschelbronn) did not reveal the presence of D, C, E, c, e, RBC antigens in the blood obtained from both patients. We performed Rh phenotyping with two sources of antisera (Diagast 251/AV.AVINEE- 59120 Loos, France and CE- Immunodiagnostika, Am Seerain 13 Germany, Eschelbronn). Positive and negative control tests were performed for each antigen according to the manufacturer’s recommendations. Based on these collective findings, we interpreted these results as being strongly suggestive of the Rhnull phenotype with a clinically significant anti-Rh29 identified in serum of the female patient whose data are shown in Table 1.

**Table 1:** The Baseline Data for Both Identified Rhnull Patients in Iran

**Type of Rare Blood ** **Group**	**Rhnull**	
Year of Identification	2013	
	**Case I**	**Case II**
Patient / Family Member	Patient	Brother
Age	43y	39y
Gender	Female	Male
Chief Complaint	Severe Anemia Following Splenectomy (Hb=3 g/dL)	Family Member
Past Medical History	Recurrent Abortion and Death Birth	-
History of Blood Transfusions	No	No
Born out of Consanguineous Marriage	Yes	Yes
ABO Blood Group	A	A
Rh (D) Type	Negative	Negative
Geographical Region	Qazvin	Qazvin
Number of Frozen Unit	None	2
Antibody Screening/ identification Test	Positive/Anti-rh29	Negative
Rh Phenotyping Test	Rhnull	Rhnull

Compatibility testing showed that serum from the female patient was non-reactive with her brother. Two units of RBC were collected from the brother within a 10-day period. She was transfused with the RBC units and never needed any transfusion since that date. Her brother donated two more units of RBC in 2014 and 2015. Cryopreservation was also utilized for future use.

## Discussion

 The Rh-deficiency syndrome was first described in 1961 by Vos^   7 ^ when a sample of blood completely failed to react with various Rh antisera. But R. Ceppellini^   8 ^ used the term "Rhnull" for the first time. To date, there are at least 43 persons belonging to 14 families with Rhnull phenotype who have been reported in the literature^   9 ^. A few Rhnull individuals were identified because of the presence of Rh antibodies in their sera, while others were detected with routine Rh phenotyping of the red cells. Interestingly, three cases were identified when the patients were investigated for determination of the appropriate blood group for blood transfusion, hemolytic anemia and abnormal red cell morphology^   10 ^.

It seems that consanguineous marriage plays an important role in the creation of this type of blood group. In the current study, our patients were siblings and were born to consanguineous parents. Antibody in immunized Rhnull individuals is considered to be "anti-total Rh" and has been given the numerical designation anti-Rh29^   9 ^. In this report, the female patient probably got immunized in pregnancy or childbirth and the antibody presented in patient’s sera reacted with all cells tested, and therefore was labeled as anti-total Rh (Anti-Rh29). But in the male patient with no history of blood transfusion, no antibody was present. 

The clinical, hematological and biological features associated with this rare disorder show that it affects the membrane integrity of red blood cells and, as evident in this report, it is associated with spherocytic hemolytic anemia, stomatocytosis and increased osmotic fragility of red cells^   9 ^. In the recent study, the male patient underwent splenectomy because of hereditary spherocytosis anemia. Further genetic studies are needed for the underlying mutation for characterization of regulator or amorphous type.

Iran has been a member of the International Society for Blood Transfusion (ISBT) working party on rare donors since 2010^   11 ^^.^ Recognition of individuals with Rhnull phenotype was reported to the working party in order to be added to the list of countries with Rhnull donors. Table 2 shows report from the ISBT Rare Donor Working Party, the 23^rd^ regional congress of the ISBT, Amsterdam, the Netherlands 2013.

**Table2:** Countries with Rare donor Rhnull phenotype

**No**	**Country ** **Name**	**Number ** **of Donors**	**ABO Typing**	**Cryopreserved**
1	Brazil	4	2 A 2 O	No Report
2	United Kingdom	1	No Report	No Report
3	China	5	No Report	No Report
4	Germany	3	No Report	11 units
5	Finland	2	No Report	No Report
6	United States	2	1 A 1 O	No Report
7	France	3	1 A 2 No Report	7
8	Iran	2	2 A	2
9	South Africa	2	1 A 1 O	No Report
10	Spain	1	No Report	No Report
11	Japan	4	No Report	No Report
12	Switzerland	1	No Report	Donor in France

Blood transfusion services might face challenges in providing compatible blood for patients with red blood cells lacking high-incidence antigens and who have made the corresponding alloantibody. A hospital blood bank should have a written protocol in place, explaining what needs to be done for rare blood request. In this event, providing blood for a patient whose antibodies reacted with RBCs of all available ABO compatible units, management of the time with quick response along with a sound clinical and laboratory judgment are necessary to save the patient’s life.

Availability of a National Rare Donor Program and support of well-trained reference laboratory personnel in a close collaboration with relevant medical team help to manage a patient’s need to a very rare Rhnull phenotype in an acceptable turnaround time. In this study, the patient’s life was saved by her brother’s compatible Rhnull blood. After consultation with his brother, he consented to be added to the list of National rare donor registry database in Iran. 

Rhnull blood group is a rare blood group worldwide that consanguineous marriage plays an important role in the creation of this type of blood group. Because of the prevalence of consanguineous marriage in Iran, it may be more prevalent in Iran compared to other regions of the world. Its incompatibility between mother and fetus can be considered as the main cause of abortion that raises the necessity of evaluation of pregnant women, especially in women with a history of miscarriage and abortion. 
